# The Diagnostic and Prognostic Value of Neurofilament Heavy Chain Levels in Immune-Mediated Optic Neuropathies

**DOI:** 10.1155/2012/217802

**Published:** 2012-12-17

**Authors:** Axel Petzold, Gordon T. Plant

**Affiliations:** ^1^Institute of Neurology, University College London (UCL), London WC1N 3BG, UK; ^2^Department of Neurology, Vrije Universiteit Medisch Centrum (VUMC), Amsterdam, The Netherlands; ^3^The National Hospital for Neurology and Neurosurgery, University College London (UCL), London WC1N 3BG, UK

## Abstract

*Background*. Loss of visual function differs between immune-mediated optic neuropathies and is related to axonal loss in the optic nerve. This study investigated the diagnostic and prognostic value of a biomarker for neurodegeneration, the neurofilament heavy chain (NfH) in three immune-mediated optic neuropathies. *Methods*. A prospective, longitudinal study including patients with optic neuritis due to multiple sclerosis (MSON, *n* = 20), chronic relapsing inflammatory optic neuritis (CRION, *n* = 19), neuromyelitis optica (NMO, *n* = 9), and healthy controls (*n* = 28). Serum NfH-SMI35 levels were quantified by ELISA. *Findings*. Serum NfH-SMI35 levels were highest in patients with NMO (mean 0.79 ± 1.51 ng/mL) compared to patients with CRION (0.13 ± 0.16 ng/mL, *P* = 0.007), MSON (0.09 ± 0.09, *P* = 0.008), and healthy controls (0.01 ± 0.02 ng/mL, *P* = 0.001). High serum NfH-SMI35 levels were related to poor visual outcome. *Conclusions*. Blood NfH-SMI35 levels are of moderate diagnostic and more important prognostic value in immune-mediated optic neuropathies. We speculate that longitudinal blood NfH levels may help to identify particular disabling events in relapsing conditions.

## 1. Introduction

An established biomarker for neurodegeneration are body fluid neurofilament (Nf) levels [1–3]. Damage to human central nervous system initiates a proteolytic cascade which causes release of Nf from neurons and axons into the extracellular fluid (ECF) [4]. Next, Nf move from the ECF into the cerebrospinal fluid (CSF) from where they diffuse into the systemic blood circulation. Therefore quantification of Nf from either body fluid permits to estimate the amount of neuroaxonal damage caused.

Cerebrospinal Fluid levels (CSF) of the Nf heavy chain (NfH SMI35) were higher in patients with a Clinically Isolated Syndrome including optic neuritis compared to control subjects and to correlate with disease activity [5] A Japanese study found the CSF NfH^SMI35^ concentration to be higher in patients with NMO (mean 0.75 ng) compared with the levels found in multiple sclerosis (MS, 0.09 ng/mL) [6]. This data is consistent with a European study [7]. Using a different analytical method, pNfH levels were however found to be comparable between patients with MS, NMO, spinal cord infarction, and controls [8]. Notably, CSF pNfH levels in this paper were also virtually absent from other conditions with known extensive axonal damage and high CSF NfH^SMI35^ levels suggesting a preanalytical or analytical problem.

Blood Nf heavy chain (NfH) levels have been shown to be elevated in patients with acute optic neuritis compared with control subjects, and its level correlates inversely with visual loss and the retinal nerve fibre thickness as assessed by retinal optical coherence tomography (OCT) [9–11].

The clinical spectrum of autoimmune ON includes Neuromyelitis Optica (NMO, Devic disease) and disease occurring as part of an Aquaporin 4 antibody spectrum (AQP4+), as well as Chronic Relapsing Inflammatory Optic Neuropathy (CRION) [12, 13]. Optic neuritis occurring as part of a multiple sclerosis spectrum (MSON) may present in exactly the same way as that occurring as part of an AQP4+ spectrum, whereas the treatment required for the first is very different to that required for the second [14]. The timing of steroid treatment in optic neuritis caused by NMO and other immune mediated optic neuropathies has been shown to be critical in the prevention of permanent retinal nerve fibre loss [15]. It is therefore of great use to identify cases of NMO from other immune mediated optic neuropathies.

This study investigated the diagnostic and prognostic value of plasma NfH^SMI35^ levels for differentiating NMO from non-NMO optic neuritis on the hypothesis that axonal loss in the optic nerve may be greater in the former.

## 2. Methods

### 2.1. Patients

Consecutive patients presenting with optic neuritis (ON) and healthy control subjects were recruited from The National Hospital of Neurology and Neurosurgery, Queen Square, St. Thomas' Hospital, and Moorfields Eye Hospital, all based in London, UK. Patients were classified into ON in the context of multiple sclerosis (MSON), CRION, and NMO as described [16, 17]. In all patients a diagnosis of optic neuritis was made clinically as described [9]. We exclude patients in whom transient visual disturbances were due to Uhthoff's phenomenon.

Serum samples were obtained by antecubital venopuncture. All samples were collected and processed at room temperature in polypropylene tubes within two hours after venopuncture. Samples were spun at 2000 g for 10 minutes. Samples were then stored in 500 uL aliquots in 1.5 mL Eppendorf tubes at −80°C.

Visual acuity (VA) was measured on Snellen charts and expressed in decimals. In cases of unilateral ON, the VA of the affected eye was recorded. In the case of bilateral ON, the VA of the worse eye was recorded. Poor vision was defined as hand movements only (6/60). National ethics permission for the study was sought by and granted to the Department of Neurology, Walton centre for Neurology and Neurosurgery, Liverpool (L9 7LJ), and informed consent was obtained from all subjects.

### 2.2. Neurofilament Test

Serum neurofilament levels (NfH^SMI35^) were measured in duplicates with the analyst being blinded to all other information using a standard in-house ELISA [18]. In brief, the mouse monoclonal antibody SMI35 was purchased from Sternberger Monoclonals Inc. This antibody is now available through Covance Research Products (Berkeley, CA, USA). Both, the secondary and tertiary antibodies were polyclonal and purchased from (Sigma, St. Louis, MO, USA; N 4142) and (DAKO, Copenhagen, Denmark, horseradish peroxidase (HRP)-labeled swine polyclonal anti-rabbit IgG). Adhering to a previously proposed nomenclature, we indicate the captured antibodies used for NfH quantification in superscript (NfH^SMI35^ for SMI35). The detection limit for the NfH^SMI35^ assay is 0.01 ng/mL. Serum NfH^SMI35^ levels above the highest value observed in the control group were classified as pathological.

### 2.3. Aquaporin 4 Test

Serum aquaporin 4 antibodies (AQP4) were tested at the Mayo Clinic laboratories by indirect immunofluorescence as described [19].

### 2.4. Statistical Analysis

All statistical analyses were performed using SAS (V9.1). For comparison of two variables the nonparametric Wilcoxon two-sample test was used. General linear model were used for comparison of more than two variables. Proportions of patients were compared using Fisher's exact test. A *P* value of 0.05 was accepted as significant.

## 3. Results

The demographic data is summarised in Table 1.

### 3.1. Diagnostic Value of NfH^SMI35^

**Figure 1:**
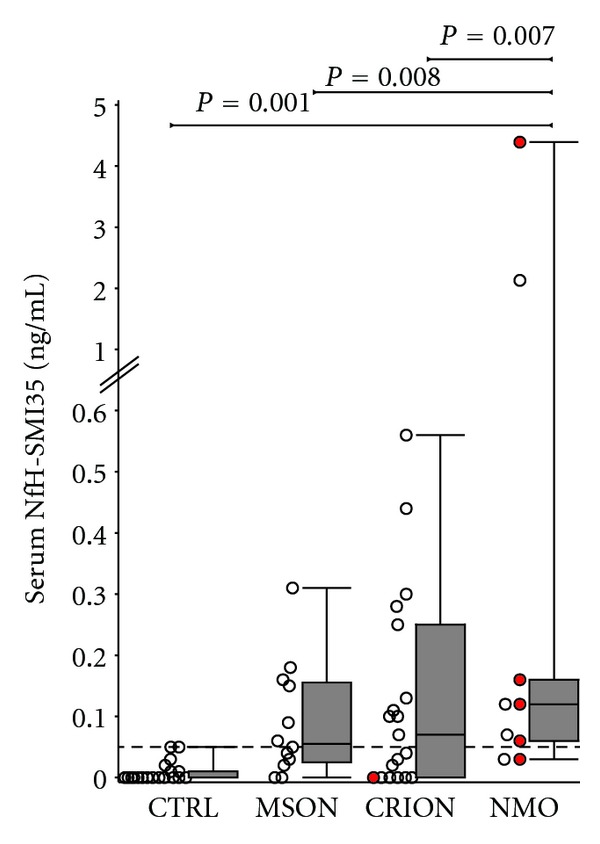
Serum NfH^SMI35^ levels are elevated in NMO compared to control subjects and other inflammatory optic neuropathies. Patients who were AQP4 seropositive are indicated in red.

Serum NfH^SMI35^ levels were significantly different between the groups (*F*_3,56_ = 4.05, *P* = 0.011). Serum NfH^SMI35^ levels were significantly higher in patients with NMO (mean 0.79 ± 1.51 ng/mL) compared to patients with CRION (0.13 ± 0.16 ng/mL, *P* = 0.007), MSON (0.09 ± 0.09, *P* = 0.008), and healthy controls (0.01 ± 0.02 ng/mL, *P* = 0.001, Figure 1).

From Figure 1 it is visible that the data was not normally distributed and serum NfH^SMI35^ levels were particularly high in two patients with NMO. For statistical rigorosity the data was therefore also analysed on a categorical level. The proportion of patients with NMO who had pathological levels (7/9) was significant larger compared to controls (0/20, *P* < 0.0001). Significance remained after the two NMO patients with particular high serum NfH^SMI35^ levels were removed (*P* < 0.0001).

Patients with MSON displayed the lowest levels of NfH^SMI35^ out of the three optic neuritis subtypes, although the difference between levels found in MSON and CRION was markedly smaller than that between these two groups and NMO on the one hand and healthy controls on the other.

### 3.2. Prognostic Value of NfH^SMI35^

**Figure 2:**
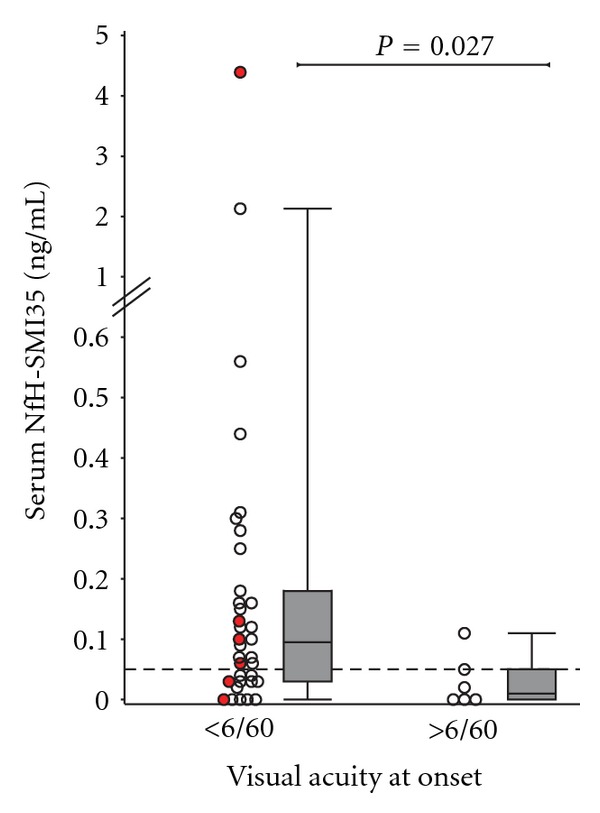
More severe loss of vision at onset is associated with higher serum NfH^SMI35^ levels.

**Figure 3:**
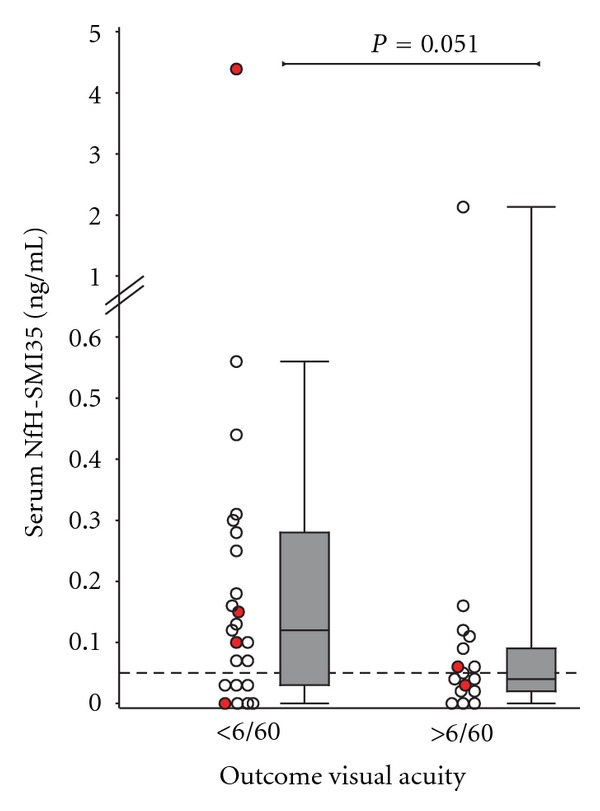
High serum NfH^SMI35^ levels are associated with poor visual outcome.

Poor VA at onset (Figure 2) and at the last follow-up visit, (Figure 3) was associated with high serum NfH^SMI35^ levels in the pooled group analysis. The NfH^SMI35^ level at the onset of optic neuritis correlated stronger with VA than did the NfH^SMI35^ level at the last follow-up visit.

## 4. Discussion

To the best of our knowledge, this is the first published study reporting the levels of NfH^SMI35^ in the serum of patients with NMO and CRION. This study suggests that serum NfH^SMI35^ levels are of more prognostic then diagnostic value in the setting of acute immune mediated optic neuropathies.

Our results are comparable to data on plasma NfH^SMI35^ levels in acute ON, where the median level of plasma NfH^SMI35^ was found to be 0.17 ng/mL in patients with optic neuritis and 0.005 ng/mL in control subjects [9].

Our findings support the concept that axonal loss following optic neuritis is more extensive in patients with NMO compared to MSON [6]. This is consistent with the clinical observation of more server loss of visual function and retinal nerve fibres in patients with NMO [20]. Interestingly, loss of retinal nerve fibres as quantified by OCT was correlated to serum pNfH levels in patients participating in the Optic Neuritis Treatment Trial [11].

The correlation of high serum NfH^SMI35^ levels with poor visual recovery in the present study further supports the argument that axonal destruction with resulting retinal nerve fibre loss may be to blame for the permanent visual deficit after the attack. We speculate that longitudinal assessment of blood NfH^SMI35^ levels may help to identify more severely disabling events in relapsing conditions. It may be interesting to find out whether or not blood NfH^SMI35^ were therefore useful to improve prognostic accuracy in patients with AQP4 seropositive ON [17, 21] or in patients with NMO in whom isolated peaks in serial antibody titres suggest active disease [22].

A weakness of this study is that we did not have systematic spinal cord MRI performed in all patients. This may be relevant because high serum NfH^SMI35^ levels may also be caused by axonal loss due to concomitant myelitis in NMO which is clinically silent. In this context it is of note that the highest serum NfH^SMI35^ levels were found in an NMO patient who was also AQP4 seropositive. In total 4/5 (80%) of the AQP4 seropositive NMO patients had serum NfH^SMI35^ levels above the highest value observed in the control cohort (0.05 ng/mL, horizontal dotted line in Figures 2 and 3). This may require further investigation of blood NfH^SMI35^ levels, particularly during an acute relapses in NMO. Of note, removal of the two NMO patients with particular high serum NfH^SMI35^ levels did not change the statistical significance of the finding. It should be mentioned that elevated serum NfH^SMI35^ levels can also be observed in other neurological and nonneurological conditions such as cardiac arrest [23], cardiac surgery [24], traumatic brain injury [25], blast injury [26], subarachnoid haemorrhage [27], stroke [28], endcarotidectomy [29], and motor neuron disease [30, 31]. None of these were present in the patients reported here. Some of these studies presented longitudinal data which showed that serum NfH^SMI35^ levels peaked early after acute injury. Therefore an important shortcoming of our study was that the exact timing of sample acquisition in relation from onset of ocular pain to venopuncture was not collected systematically. This is an important point because onset of ocular pain may precede onset of visual loss and should be considered as time of onset in future studies. We cannot exclude the possibility that patients presenting with more severe loss of vision were recruited at an earlier time point in their disease which could have skewed the data.

Another shortcoming of the study was that all patients were recruited through a busy routine UK NHS clinic. Therefore the timing of investigations and followup was less systematic than what would have been desirable and missing data limits the power of the present study. On the other hand the data presented may be more reflective for a hands on day-to-day neurological practise than a randomised clinical trial setting. Likewise, a limitation of the study is that assessment of low contrast VA was not performed [32]. Because recovery of low-contrast VA and colour vision following optic neuritis is poorer compared to high contrast VA, correlation analyses of serum NfH levels with these measures should be performed in future studies.

After this study was completed a number of methodological papers appeared in the literature showing differences in the analytical sensitivity of a range of AQP4 tests [33]. Using some of the more sensitive tests may have increased the proportion of AQP4 positive NMO cases in the present study.

In conclusion, blood NfH^SMI35^ levels are of moderate diagnostic and reasonable prognostic value in patients presenting with an immune-mediated optic neuropathy.

## Figures and Tables

**Table 1:** Patient characteristics. The median (numbers) are presented.

	CTRL	MSON	CRION	NMO
*N*	20	28	19	9
Age	34	33	45	29
Followup	n/a	5	65	67
VA baseline	≥1	0.1	0.008	0.01
VA outcome	n/a	0.67	0.1	0.33
NfH (ng/mL)	0.00	0.055	0.07	0.12

F: female, M: male, VA: visual acuity, CTRL: control subjects, MSON: multiple sclerosis optic neuritis, CRION: chronic relapsing isolated optic neuropathy, NMO: neuromyelitis optica.
